# Climate change-induced degradation of expert range maps drawn for kissing bugs (Hemiptera: Reduviidae) and long-standing current and future sampling gaps across the Americas

**DOI:** 10.1590/0074-02760230100

**Published:** 2024-09-23

**Authors:** Vaughn Shirey, Jorge Rabinovich

**Affiliations:** 1 University of Southern California, Department of Biological Sciences, Marine and Environmental Biology Section, Los Angeles, CA, United States; 2 Georgetown University, Department of Biology, Washington, DC, United States; 3 Universidad Nacional de La Plata, Centro de Estudios Parasitológicos y de Vectores, La Plata, Argentina

**Keywords:** climate change, bias, biodiversity knowledge, kissing bugs, range maps, Triatominae

## Abstract

**BACKGROUND:**

Kissing bugs are the vectors of *Trypanosoma cruzi*, the etiological agent of Chagas disease (CD). Despite their epidemiological relevance, kissing bug species are under sampled in terms of their diversity and it is unclear what biases exist in available kissing bug data. Under climate change, range maps for kissing bugs may become less accurate as species shift their ranges to track climatic tolerance.

**OBJECTIVES:**

Quantify inventory completeness in available kissing bug data. Assess how well range maps are at conveying information about current distributions and potential future distributions subject to shift under climate change. Intersect forecasted changes in kissing bug distributions with contemporary sampling gaps to identify regions for future sampling of the group. Identify whether a phylogenetic signal is present in expert range knowledge as more closely related species may be similarly well or lesser understood.

**METHODS:**

We used species distribution models (SDM), specifically constructed from Bayesian additive regression trees, with Bioclim variables, to forecast kissing bug distributions into 2100 and intersect these with current sampling gaps to identify priority regions for sampling. Expert range maps were assessed by the agreement between the expert map and SDM generated occurrence probability. We used classical hypothesis testing methods as well as tests of phylogenetic signal to meet our objectives.

**FINDINGS:**

Expert range maps vary in their quality of depicting current kissing bug distributions. Most expert range maps decline in their ability to convey information about kissing bug occurrence over time, especially in under sampled areas. We found limited evidence for a phylogenetic signal in expert range map performance.

**MAIN CONCLUSIONS:**

Expert range maps are not a perfect account of species distributions and may degrade in their ability to accurately convey distribution knowledge under future climates. We identify regions where future sampling of kissing bugs will be crucial for completing biodiversity inventories.

Kissing bugs (Hemiptera: Reduviidae: Triatominae) are a largely hematophagous group of insects that are all potential vectors for *Trypanosoma cruzi* (Trypanosomatidae: *Trypanosoma*), a parasite that causes Chagas disease (CD), a potentially life-threatening condition that can cause long-term heart disease and damage to other organs. The parasite is typically passed into the human bloodstream after the insect completes a blood meal, and defecates on the hosts’ skin, passing the *T. cruzi* parasite from the feces into the host’s circulatory system.^1^ Importantly, with increasing habitat fragmentation and climate change, it is projected that several kissing bug species may become epidemiologically more relevant, both in the endemic range of many species and in new areas that may become suitable to kissing bugs in the future under changing environmental conditions.^2^ ^,^ ^3^ ^,^ ^4^ ^,^ ^5^ ^,^ ^6^

Being important disease vectors, kissing bug species have been previously examined through the lens of species distribution modeling (SDM) at both local and broader geographic scales.^7^ ^,^ ^8^ ^,^ ^9^ ^,^ ^10^ ^,^ ^11^ Kissing bugs are largely Neotropical in their distribution with some species extending their range into the southern Nearctic. Their presence seems to be largely driven by climatic tolerances^5^ ^,^ ^6^ ^,^ ^12^ ^-^ ^17^ as well as by the presence of suitable hosts from which to take a blood meal.^18^ ^,^ ^19^ ^,^ ^20^ ^,^ ^21^ ^,^ ^22^ For example, several previous works have demonstrated that before the advent of SDMs, the ecological factors considered most important for influencing the distribution of kissing bugs were temperature and humidity.^23^ Since then, it was recognized that some species endure more or less variation while others are harmed by the same fluctuations which was corroborated by SDMs analyses.^6^ ^,^ ^17^ ^,^ ^24^ ^,^ ^25^ Temperature in particular is important for thermal preference^26^ ^,^ ^27^ ^,^ ^28^ as well as host finding, feeding, reproduction, and development.^29^ ^,^ ^30^ ^,^ ^31^ ^,^ ^32^

The early detection of the dispersal and/or range shifts of triatomine species to new areas (especially those populated by humans) is critical for assessing potential future public health threats.^3^ ^,^ ^33^ ^,^ ^34^ ^,^ ^35^ ^,^ ^36^ Opportunistically sampled data such as those from museum collections can provide a historical basis for triatomine species ranges; additionally, community science is playing an increasing role in triatomine species early detection,^37^ ^,^ ^38^ ^,^ ^39^ as has been the case for invasive insect species elsewhere.^40^ Thus, it is important to establish a baseline assessment of where the knowledge of triatomine occurrence is most complete, and to examine which regions are under-sampled in order to target those areas in future sampling.

Given the importance of kissing bugs to public health, we sought to assess the current state of our knowledge of kissing bug occurrence from publicly available datasets. These datasets include DataTri^41^ a curated database of triatomine occurrence records, and records from the Global Biodiversity Information Facility (GBIF),^42^ which include “Research Grade” records from community science platforms such as iNaturalist as well as Integrated Digitized Biocollections (iDigBio) and Symbiota Collections of Arthropods Network (SCAN).^43^ Previous work on inventory completeness and the assessment of bias in insect data has demonstrated that regions experiencing drastic climate change are likely to be under sampled^44^ as sampling efforts typically focus on regions of high human population density or at the interface of anthropogenic infrastructure (*e.g.*, roads and recreational trails).^44^ ^,^ ^45^ ^,^ ^46^ Recent research has shown that these biases not only occur in triatomine sampling schemes,^47^ but also increase in magnitude over time.^46^

We expect kissing bugs to exhibit similar trends, mainly because ― being vectors of a human disease ― most of the sampling efforts are made at or near domiciliary rural sites^48^ and because their wild habitats are difficult to sample.^49^ Here we focus on how expertly drawn range maps compared to maps produced by species distribution models. In particular, we highlight how range maps may degrade in their ability to convey accurate information about species occurrence, especially in light of forecasted climate change. Finally, we performed an analysis of several kissing bug species, their probability of occurrence over time and gaps in species inventories to identify priority regions for the future sampling of this group.

## MATERIALS AND METHODS

*Species range maps and occurrence data* - Species range maps were obtained from the triatomine geographical Atlas by Carcavallo et al.^50^ These maps cover the breadth of potential kissing bug distributions throughout the neotropics and adjacent regions including the southern United States. Those maps were drawn by hand by the senior author (Dr Rodolfo Carcavallo) and there was no explanation for the methodology or procedures used in their drawing. Those range maps were scanned and digitized, recording the areas of presence at a scale of 0.1 x 0.1 coordinates degrees; so, our working data were a set of latitude/longitude coordinates representing those range maps, both graphically and as data in a spreadsheet. The author of the Atlas was an excellent “expert” in triatomines, collected tirelessly in all of the Americas personally, and was an inexhaustible collector of bibliography (his atlas is based not only in his personal experience but also on hundreds of locations cited by many other researchers). There were 115 triatomine species included in this original atlas; however, due to taxonomic revisions since the creation of the Atlas, there are now 112 valid species in the atlas.^51^ ^,^ ^52^ ^,^ ^53^

Confirmed occurrence records for triatomine species were gathered from DataTri,^41^ the Global Biodiversity Information Facility (GBIF),^42^ the Integrated Digitized Biocollections (iDigBio), and the Symbiota Collections of Arthropods Network (SCAN) (see Supplementary data for a list of collections accessed). These occurrence records were then filtered to include only species for which we had range map information and in which the point occurrence intersected with the range map for that species for our inventory analysis. We retained all records for our SDM and expert score assessments. Additionally, we only included records of kissing bugs observed/collected from 1910-2021, further partitioning these into two distinct datasets for our analysis. All subsequent described analyses were conducted in R v. 4.2.1.^54^

*Inventory completeness analysis* - Expected richness was calculated for 100×100 km square cells by layering expert range maps and counting the number of overlapping ranges per square cell: observed richness was calculated by counting the number of unique species from the filtered occurrence data for each grid cell. Thus, if expert ranges for three kissing bug species overlapped in a given cell, the expected richness was three. The ratio was calculated using the following equation:

$$Inventory\;completeness=\frac{(Observed\;richness\;from\;ocurrences)\;}{\left(Expected\;richness\;from\;range\;maps\right)}$$

A ratio of observed richness to expected richness was calculated from these two values and fell between zero (no records of kissing bugs for which we had range maps) and one (complete recording of expected kissing bug richness based on range maps). This approach for inventory completeness has been performed elsewhere.^44^

*Species distribution models* - We ran species distribution models using Bayesian Additive Regression Trees (BARTs) via the package “embarcadero”.^55^ Like other machine-learning methods, BARTs compute a binary representation (using a logit-link function where applicable) of habitat suitability for a species (see Carlson^55^ for more detail on the algorithm). In addition, BARTs have the added benefit of reduced overfitting problems that can be common in decision tree approaches.^56^ We assessed the performance of our SDMs by examining the average area under the receiver-operator curve (AUC) across five top models per species. AUC indicates the overall performance of the model with respect to true and false positive rates.^57^ Values above 0.5 indicate the model is performing better than random chance with respect to predictive capacity. The top models for each iteration of the BART procedure were selected using the highest training true skill statistic (TSS).^58^ The AUC and TSS scores for all discovered models can be found in the Supplementary data.

We required species to have at least 25 unique occurrence records (from the period prior to 1999) to be considered for distribution modeling. Unfortunately, this eliminated *Triatoma brasiliensis* from our pre-1999 analysis; however, given its epidemiological importance, we included it in the post-1999 analysis (where it meets our minimum requirement). Random background, or pseudoabsence, points were generated such that the number of pseudoabsence points was equal to the number of occurrence records used for modeling. Five identically specified models were run per species to obtain an average probability of occurrence.^59^ We used the Bioclim dataset for environmental predictors in our model.^60^ In the past, kissing bug distributions have been modeled using the full Bioclim dataset, with the variables BIO4 (Temperature Seasonality), BIO5 (Maximum Temperature of the Warmest Month), BIO6 (Minimum Temperature of the Coldest Month), BIO13 (Precipitation of the Wettest Month), BIO14 (Precipitation of the Driest Month), and BIO15 (Precipitation Seasonality) found to be highly predictive of kissing bug occurrence in the past.^6^ We used correlation plots to identify similar variables for inclusion in our present study to reduce issues with multicollinearity. In the case of purely predictive approaches, issues such as multicollinearity are not as relevant;^61^ ^,^ ^62^ however, we have provided correlation plots highlighting the correlation between BIOs for each species in our analysis in the Supplementary data. Studies that aim to infer the specific, causal influence of environmental factors on distributions should more explicitly consider multicollinearity among other signals of potential confounders. Finally, we partitioned our occurrence dataset into data that were collected before 1999 (to remain congruent with when the expert range maps were drawn); and data from across all periods (to examine how well models conditioned on data collected both pre- and post-1999 agreed with expert range maps). We used a 2.5-minute resolution of the Bioclim dataset across all analyses. Full specifications of the models as well as AUC diagnostics can be found in Supplementary data (Table I) (pre-1999 model diagnostics) and Supplementary data (Table II) (all occurrence model diagnostics). We also include all model files as an additional Supplementary data to this work. We used a minimum bounding box which fully encompassed both the occurrence records and expert range maps with a 500-kilometer buffer zone as the calibration area for the model. We used this minimum bounding box to constrain the analysis and reduce the potential that more global areas of calibration might have on predictions,^63^ ^,^ ^64^ including the buffer zone to allow for projections to potentially suitable environmental spaces that kissing bugs could plausibly occur but have not been explicitly sampled.

*Expert score analysis* - Following the construction of our SDM, we used the package, “expertscore” to compute our metrics for expert range maps.^65^ Expert scores close to one indicate a high congruence between expert range maps and species distribution models while values close to zero indicate that the expert map is no more predictive of species distribution than a null model map. Expert scores less than zero indicate that the null model map has greater predictive accuracy than the expert range map. In our case, the null model map was the minimum quadrilateral polygon that encompasses both the expert range map and the point occurrence data with a buffer of 500 km. Following the calculation of expert scores, we compared performance of the pre-1999 and all occurrence record analysis to each other and from the current time period into 2100 using a two-sided Wilcox test with a two-sided alternative hypothesis. We conducted this test because our expert score data did not meet assumptions of normality of residuals when conducting a t-test. These comparisons were made across datasets to assess the influence that additional occurrences may have had on the stability of expert range maps constructed from occurrences pre-1999.

Using our distribution models, we forecasted each of the species’ ranges into the periods 2041-2060, 2061-2080, and 2081-2100 under RCP 8.5 (GCM ACCESS-ESM1-5, 2.5-minute resolution) to assess the rate at which expertly drawn range maps become less/more reliable over time according to their expert scores. Model transfer to future climatic conditions was performed using the “*predict*” function in the R package “raster”^66^ with our BART models that were trained using current climatic conditions and occurrences. This allowed us to project species responses to the current environment onto forecasted environmental conditions. We also intersected the average differences in SDM predicted occurrence probabilities with our inventory completeness analysis to assess how many areas forecasted to increase in overall mean kissing bug occurrence probability (across all species) overlapped with regions that currently have little to no publicly available kissing bug data (*i.e.*, large sampling gaps). In doing this we generated a map which shows where kissing bug probability of occurrence is likely to increase intersected with where the current knowledge gaps (represented by inventory completeness) occur. We split these metrics into four equally sized partitions representing the shift in kissing bug occurrence probabilities (low to high) and the completeness of contemporary inventories (complete to incomplete). These categories were used to produce a bivariate map highlighting where future sampling might be prioritized.

Finally, we aimed to test if expert scores and degradation/improvement of these scores over time exhibited a phylogenetic signal across a tree of kissing bugs obtained from Ceccarelli et al.^67^ Several of the species in our analysis were not mapped onto an existing phylogenetic tree of kissing bug species and so we pruned those tips from our tree and subsequent analyses. This resulted in 27 species for the pre-1999 scores and 28 species for the scores using all available occurrence data. We tested for phylogenetic signal among expert scores and the magnitude/direction of expert score shifts across our tree using Blomberg’s K, K-star,^68^ and Pagel’s λ.^69^ Blomberg’s K and K-star values closer to or greater than 1.0 are indicative variance in the trait value being distributed among clades while values less than 1.0 are indicative of the variance being distributed within clades (*i.e.*, closely related species do not resemble one another).^68^ Pagel’s λ values close to 1.0 indicate a high correlation between species traits equal to Brownian motion.^69^ We used the package “phylosignal” to calculate these metrics.^70^

## RESULTS

**Fig. 1:**
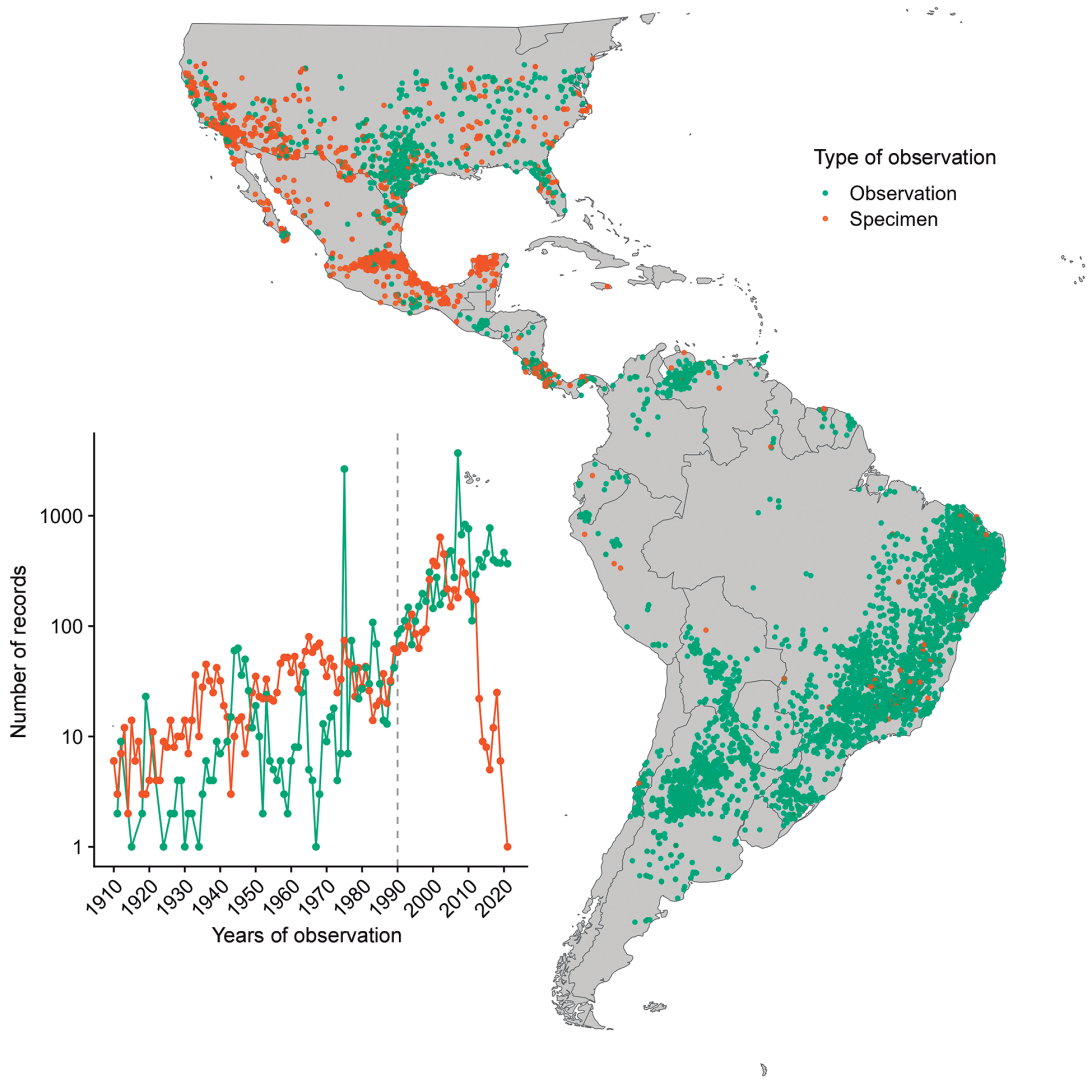
point occurrence data and years of observation (inset line plot) colored by each record’s ‘basisOfRecord’ field (of either museum specimens or human observations). A random sampling of 50% of the occurrences (*n* = 13,641) are illustrated on the map to avoid overplotting. Green = observation; Orange = collected specimens.

**Fig. 2:**
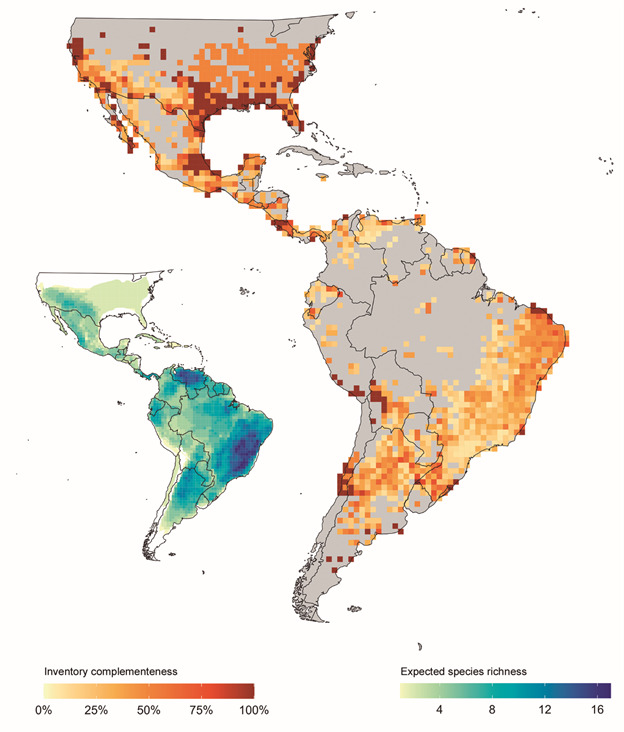
kissing bug inventory completeness at the 100×100 km spatial resolution (darker colors indicate greater inventory completeness). The inset map indicates expected richness based on overlapping expert range maps (darker colors indicate greater expected kissing bug species richness).

Records for kissing bugs are scarce, especially in remote and sparsely populated regions, particularly in the Amazon River Basin and northern Mexico (Figs 1-2). With respect to inventory completeness, several regions of low completeness emerged including southern Brazil, Uruguay, Peru, Ecuador, Colombia, Venezuela, Guyana, Suriname, and French Guiana. Notably, areas in close proximity to major cities such as Santiago, Chile; Córdoba, Argentina; Mexico City, Mexico; and San Diego/San Antonio, United States demonstrates a high density of occurrence records. In contrast, however, many regions have no single digitized occurrence record for a kissing bug species (*e.g.*, much of the Amazon River basin). Overall, our SDMs performed well in their ability to predict current kissing bug distributions according to AUC metrics ENT#091;Supplementary data (Tables I, II)ENT#093;.

**Fig. 3:**
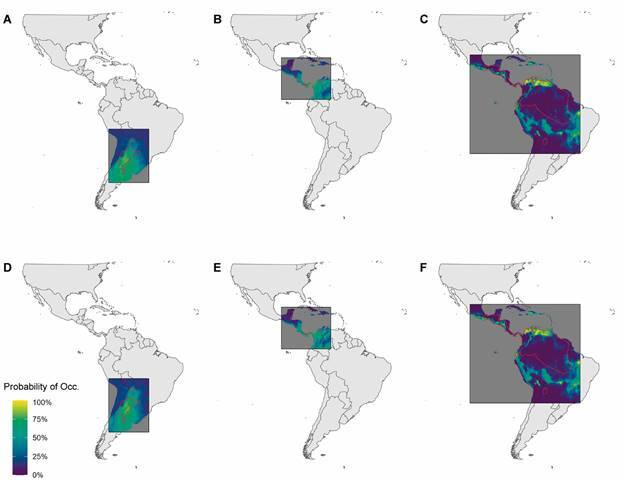
agreement between expert range maps (drawn in 1999 and shown here as red polygons) and species distribution model (SDM) output produced from only occurrence records collected before 1999 (A ,B, C) and occurrence records collected in all time periods (D, E, F). Only three species are highlighted here. Species and corresponding scores, from left to right pairs of panels, are (A, D) *Triatoma delpontei* (0.53, 0.60); (B, E) *Rhodnius pallescens* (0.54, 0.58); and (C, F) *Rhodnius prolixus* (0.51, 0.52).

**Fig. 4:**
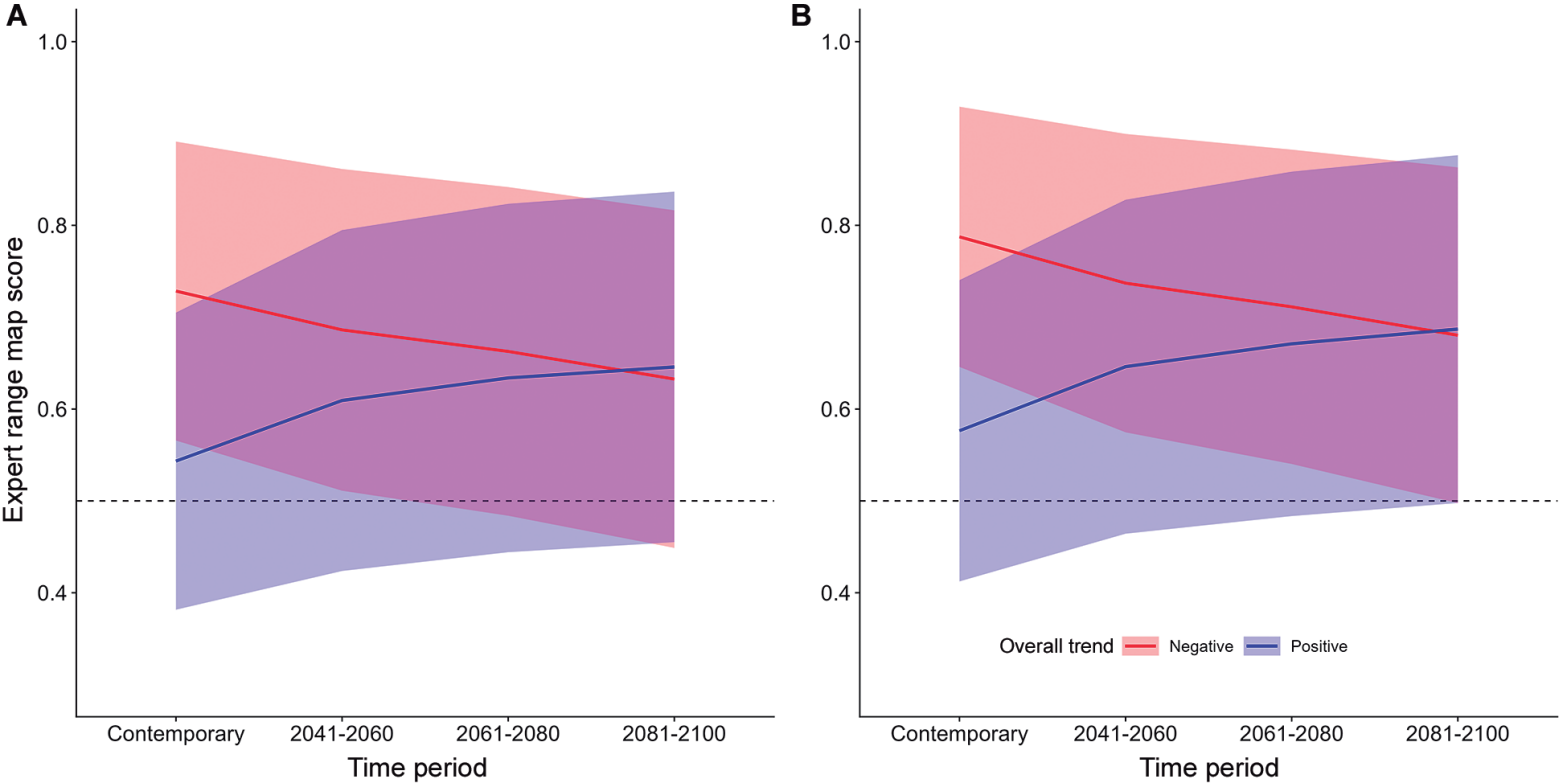
degradation of expert ranges maps over time under climate change scenario RCP 8.5 into 2041-2060, 2061-2080, and 2081-2100 (Global Circulation Model: ACCESS-ESM1-5, 2.5-minute resolution) based on species distribution projections from (A) the only pre-1999 occurrence record model and (B) the full occurrence record model. Species are summarized into two groups, declining expert scores over time (red) and increasing export scores over time (blue). The shaded area represents on standard deviation of variation around the mean trend line.

Expertly drawn range maps (constructed pre-1999) varied in their level of agreement with species distribution models constructed from occurrence data in the same time period (Figs 3A, B, C, 4, Supplementary data - Table III). When we included additional occurrence records collected post-1999, the agreement between expertly drawn range maps and SDM improved slightly overall ENT#091;Fig. 4, Supplementary data (Table IV)ENT#093;. In total, we obtained enough information to model 32 (pre-1999) and 33 (all occurrence) species of kissing bugs. We used the full dataset of either 32 or 33 species for all subsequent analyses. Among all species modeled in both the pre-1999 and all occurrence record frameworks, *Panstrongylus geniculatus* expressed the least agreement (0.24 and 0.29) with its expertly drawn range map. *P. megistus* and *T. guasayana* expressed the best agreement in the pre-1999 analysis (0.91 and 0.89) and all occurrence analysis (0.95 and 0.92) respectively. The average contemporary expert map score was 0.68 ENT#091;standard deviation (SD) +/- 0.18ENT#093; for the pre-1999 models and 0.73 (SD +/- 0.17) for the models using all occurrence records (Fig. 4). The distribution of expert scores across species from both datasets were not significantly different according to the Wilcox test (W = -423, p-value = 0.171).

Over time, most expertly drawn range maps degrade in their ability to convey accurate information about kissing bug species distributions under a “business-as-usual” carbon emissions scenario (RCP 8.5) (Fig. 4). On average, expert range maps scores from now through the 2100s shifted by -0.046 (SD +/- 0.11) and -0.054 (SD +/- 0.13) for the pre-1999 models and all occurrence models respectively (Fig. 4). There was no significant difference between the shift in expert score across the pre-1999 and all occurrence models (W = 553, p-value = 0.75). Expert range maps for *T. lecticularia* (-0.35) had the greatest decline in being able to convey information about kissing bug occurrence into the 2081-2100 time period from the pre-1999 model. When examining declines in the ability of expert range maps to convey accurate information about species using all available occurrence data, *T. lecticularia* (-0.36) and *T. brasiliensis* (-0.35) emerged as range maps with sharply declining accuracy into 2081-2100. Notably, several species range maps became more accurate over time including *R. pallescens* (+0.26) and *T. rubrofasciata* (+0.12) (pre-1999 models); and *R. pallescens* (+0.25) and *E. cuspidatus* (+0.16) (all occurrence models).

**Fig. 5:**
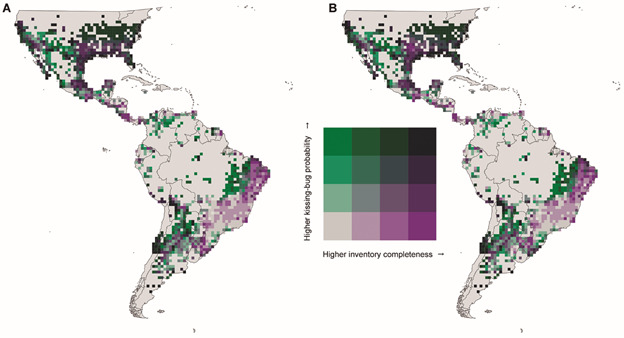
potential regions in which to better sample kissing bug occurrence across the Americas. Targeted regions of sampling (green axis) between the (A) pre-1999 occurrence and (B) all occurrence models mostly overlap. Please note that this figure does not include measures of disease risk, simply projected distributions, and existing sampling gaps. Grey-green scale indicates higher probability of future kissing bug occurrence in 2080-2100 (green indicates higher probability) while the purple-grey scale indicates inventory completeness (purple indicates higher inventory completeness).

When examining the mean occurrence probability shift into 2081-2100 for all kissing bugs over our time period of inference, regions experiencing notable sample gaps today are also regions forecasted to increase in the occurrence probability of kissing bugs on average (Fig. 5). Notably, regions already experiencing sampling gaps with respect to inventory completeness such as the eastern Amazon River Basin, parts of Venezuela, central Mexico, the Argentina/Paraguay/Bolivia border, and the Mexico/United States border were identified as key regions where average kissing bug occurrence is forecasted to increase and notable contemporary sampling gaps occur.

Across the kissing bug tree, we found generally low support for expert scores/change in expert scores being related strongly to phylogeny. In our pre-1999 analysis, Blomberg’s K and K-star were estimated to be smaller than 1.0 (0.26 ENT#091;p = 0.50ENT#093; and 0.31 ENT#091;p = 0.49ENT#093; respectively). Pagel’s for this set of expert scores was estimated to be 0.31 (p = 0.15). The estimated shift in expert range map score also exhibited low phylogenetic signal for the pre-1999 analysis with Bloomberg’s K at 0.399 (p = 0.16), but K-star was estimated at 0.58 (p = 0.029) indicating stronger phylogenetic signal in the shift in expert score from now into 2100. Pagel’s λ for the shift in expert score metric in this analysis was estimated to be 6.2 x 10^-5^ (p = 1.0). In our analysis of expert score using all available occurrence information Blomberg’s K and K-star were estimated to be 0.31 (p = 0.33) and 0.33 (p = 0.49) for the contemporary score respectively. Pagel’s λ was estimated to be 0.35 (p = 0.03). With respect to the change in expert score from now into 2100, Blomberg’s K and K-star were estimated to be 0.36 (p = 0.27) and 0.54 (p = 0.09) respectively. Pagel’s λ was estimated to be 4.5 x 10^-5^ (p = 1.0). A full table including all scores can be found in Supplementary data (Table V). This pattern of detecting both low phylogenetic and slightly elevated phylogenetic signal across metrics may be, in part, due to the smaller size of the phylogeny we used in this analysis.^71^

## DISCUSSION

Despite their importance for public health, available information on kissing bug occurrence is shockingly sparse, mainly from regions that include much of the Amazon River Basin and parts of Mexico, Central and Northern South America (Figs 1-2). Even in regions with relatively high densities of occurrence records such as those in south-eastern Brazil and parts of Venezuela, inventory completeness for the group is low (Fig. 2), and this is even more surprising given that those areas have a high-density rural as well as metropolitan centers. In concordance with previous research, our expert range maps indicate high species richness for kissing bugs in Brazil^7^ and across northern South America.^6^ Specifically, Gurgel-Gonçalves et al.^7^ in particular demonstrated high species richness along the Atlantic coast of Brazil which we also find from overlaying expert range maps (Fig. 2). We did not find high richness in the states of Ceará, Rio Grande do Norte, Paraíba, Pernambuco, and related regions, but this may be due to the range maps having been constructed from fewer data points pre-1999.

Our phylogenetic analysis of expert scores revealed low phylogenetic signal among contemporary scores (and, for that matter, scores across other projected time periods) ENT#091;Supplementary data (Table V)ENT#093;. We assumed that as all species belong taxonomically to one subfamily, the ability to accurately convey a range map may be similar for closely related species (*e.g.*, closely related species may be similarly detectable or share a similar niche space).^72^ ^,^ ^73^ ^,^ ^74^ Similarly, we expected that there may be a phylogenetic signal in the change in expert score over time given that closely related species may respond similarly to changing climatic conditions. This was not the case in our analysis as Blomberg’s K and Pagels λ estimates were quite low ENT#091;Supplementary data (Table V)ENT#093;. Testing expert scores and their forecasts shifts over time using a larger sample of kissing bug species and a tree with additional tip reflecting those species may help resolve this apparently conflicting evidence for phylogenetic signal in scoring.

Some of the same locations with notable sampling gaps (Fig. 2) correspond with where kissing bugs are expected to increase in their occurrence probability into 2081-2100 under a “business-as-usual” climate change scenario (Fig. 5). This may make these regions, especially the eastern Amazon River Basin, parts of Venezuela, central Mexico, the Argentina/Paraguay/Bolivia border, and the Mexico/United States border, important to sample in the future both to confirm the results of our species distribution forecasts but also to detect emerging and important vector species on the move. Baseline expert range map scores may also be impacted by misidentifications of historical material, as may be the case with the *Rhodnius* species group, where the expert range map indicates a wide distribution of *R. proxilus* (Fig. 3), but many specimens may have actually been *R. neglectus* based on a more recent morphometric analysis.^75^

Several triatomine species have been recorded to have been expanding both in range and in habitat type: *e.g.*, *P. geniculatus*, considered until recently a sylvatic triatomine^50^ that fed almost exclusively on the nine-banded armadillo (*Dasypus novemcinctus*), is now being found in human habitations in Colombia,^11^ ^,^ ^76^ Brazil,^77^ ^,^ ^78^ and Venezuela.^79^ However, to determine if the cause of such distribution/habitat changes can be attributed to climatic or non-climatic factors is extremely difficult, for usually several factors are concomitant in determining these geographic/habitat changes. Catalá^80^ has shown that in *T. infestans* the most attractive habitats for dispersing bugs would be those at short distance, with high CO_2_ emission and strong IR radiation, indicative of host presence (within the domestic habitat goat corrals may be the most attractive habitat to disperse); additionally, dispersal would be favored in periods of low atmospheric water saturation when IR perception is highest.^80^ Furthermore, Baines et al.^81^ have suggested that phenotype-by-environment interactions strongly influence dispersal of populations. On the other hand, transient changes in dispersal are common in many species undergoing range expansion, and may have major population and biogeographic consequences.^82^ In the case of triatomines, inter-specific competition seems to be one of those non-climatic factors; *e.g.*, (a) *T. rubrovaria* in Brazil is found in the wild in rocky habitats, but between 1975 and 1997, a growing domiciliary and peridomiciliary invasion of *T. rubrovaria* has taken place since the control of *T. infestans*,^83^ an effect determined by inter-specific competition,^84^ (b) Abrahan et al.^85^ found that, after insecticide spraying, the sylvatic triatomines *T. guasayana*, *T. eratyrusiformis*, *T. garciabesi*, and *T. platensis*, not targeted by insecticide spraying, were captured simultaneously within peri-domestic areas and showed higher house invasion pressure than *T. infestans*, and (c) in Venezuela, *P. geniculatus* has been recorded to be in a process of domiciliation as a result of the control of *R. prolixus*, suggesting a competition for resources;^79^ maybe this range dispersal and habitat change of *P. geniculatus* explains why this species showed in our analysis the least agreement (0.23 and 0.28) with the expertly drawn range map. On the other hand, *T. infestans* in itself is an example of the complexity of factors affecting the geographic/habitat occupation of triatomines, mainly because it has expanded its range into rapidly developing cities of Latin America,^86^ but also restricting its range due to the intensive use of insecticide campaigns to control this vector species. Reductions in the geographic area of these important vector species have been quantified in the literature after such vector control programs have been implemented. For example, Ribeiro Jr et al.^87^ demonstrated a reduced area of occurrence for *P. megistus* and *T. infestans* but increases in *T. sordida* and *T. pseudomaculata* after control programs were implemented in Bahia, Brazil.^87^ Further examples of the influence of control campaigns on kissing bug distributions are: (a) In 1997 Uruguay was the first country to receive the International Evaluation Commission certification for achieving the interruption of *T. cruzi* transmission, through the total elimination of *T. infestans* populations,^88^ and a similar certification was given to Brazil in 1999;^89^ these two cases of human interventions impinge on the disappearance of *T. infestans* from very large regions; (b) however, there is another human intervention that acts opposite to the point above: the passive dispersal of *T. infestans* carried by people (and their domestic animals);^90^ further, Abrahan et al.^91^ consider that passive dispersal is one of the most frequent ways of spreading for triatomines over large areas. Passive dispersal of *Rhodnius* spp. by birds has also been observed.^92^ To make things more complex, Richer et al.^93^ showed by means of the detection of restricted gene flow between close but distinct sylvatic sites, that wild *T. infestans* does not disperse by flying at high altitude (2,750 meters above sea level). Recently, genomic techniques have helped to better understand the dispersal and habitat adaptation in triatomines; *e.g.*, Hernandez-Castro et al.^94^ have shown that *R. ecuadoriensis* shares outlier loci consistent with local adaptation to the domestic setting, which mapped to genes involved with embryogenesis and saliva production; and in the case of *T. infestans* Panzera et al.^95^ showed that the ribosomal patterns are associated with a particular geographic distribution, and that chromosomal markers allowed to detect the existence of a hybrid zone occupied by individuals derived from crosses between two chromosomal groups.

Importantly, we want to highlight the potential risks of inferring the direct transferability of our SDMs for kissing bugs through time. Non-stationarity is likely present in many biological populations.^96^ In other words, the effect of a climatic factor such as temperature may not be consistent across a species’ range due to underlying biological variation. This problem may be exacerbated in species with fairly large distributions that likely experience and have locally adapted to a variety of climatic conditions across their range.^96^ Additional uncertainty in our future projections may also come from the fact that we used a single RCP scenario, assuming a “business-as-usual” carbon emissions trajectory and one global circulation model. This scenario and its interaction with global circulation models may not entirely convey future conditions should human development or unforeseen climate effects take place, especially those farther out from the present. Future work in this sphere could examine the impact that these global models would have on inference regarding species ranges and expert range maps, especially for more fine-scale analyses than presented here.^97^ Further, we did not include information in our distribution models about land-cover or human/livestock population densities, instead opting to focus on climate change, but these factors may also contribute strongly to the distribution of kissing bugs.^1^ ^,^ ^19^ ^,^ ^20^ ^,^ ^21^ When considering these forecasted areas of sampling, it will be important to account for geographical spaces that kissing bugs could or could not occupy in relation to the environmental spaces predicted by our distribution models, for example in spaces that are hard to reach through dispersal. Finer scale study should explicitly incorporate this distinction.^98^ Additionally, SDMs are likely to capture the realized rather than fundamental niche of a species^99^ which may mean that our future projections are merely projections of the realized niche space which could change over time due to a myriad of factors. The temporal transfer of niche models to future conditions should be treated with caution as truly novel environments could indeed be suitable for species occurrence in addition to errors of environmental omission based on the extent of the calibration area.^100^ ^,^ ^101^ Risks of over- and underpredicting are possible considering that projections to future scenarios may include falsely suitable regions and decisions made in modelling may lead to partial niche characterization. Finally, although closely related kissing bug species may have similar niches, they may not respond to changing climates in the same or similar way. Further study should aim to disentangle the roles of geography and evolution in driving responses to future climate change in the region. Thus, we encourage that our future projections are considered in the context of these omissions and modeling decisions.

Finally, we enthusiastically encourage the continued collection and monitoring of kissing bug distribution data which will undoubtable serve as a useful validation tool for this and future analyses. Early surveillance programs including those in Brazil^83^ as well as the importance of community-science programs for the early detection of kissing bug occurrence are critical for educating and preventing potential outbreaks of CD.^102^ ^,^ ^103^ Such programs will likely become more popular and better at identifying novel occurrences of kissing bugs, especially with the advent of artificial intelligence tools like computer vision, which are already being developed to catalogue and identify kissing bug species from cellphone and other photography.^103^ ^,^ ^104^ ^,^ ^105^ ^,^ ^106^ ^,^ ^107^ ^,^ ^108^

Kissing bugs are an increasingly important group of vector insect species in the Americas. Here, we have demonstrated that our current understanding of their distributions via expert range maps is quite variable at the species level and that, on average, the ability of these expert range maps to convey accurate information about kissing bug distributions will likely decline under a “business-as-usual” carbon dioxide emissions scenario. Further, regions that are currently under-sampled for kissing bugs may also be regions that are increasing in climatic suitability for many species. With increasing human development and habitat fragmentation, interactions between kissing bugs and their hosts (both human and non-human) is quite complex: *e.g.*, Ceballos et al.^109^ showed that massive deforestation around villages or selective extraction of older trees in the Dry Chaco in Argentina, has led to reductions in opossum abundance jointly with increases in foxes and skunks, leading to a dramatic decrease of *T. cruzi* infection in wild reservoir hosts, but may be on the rise in urban and suburban habitats, despite the State and community triatomine control activities. As an important vector of CD, we should make a concerted effort to accurately and publicly document the occurrence of this important insect group well into the future.
