# Supplementary material for: Climate change-induced degradation of expert range maps drawn for kissing bugs (Hemiptera: Reduviidae) and long-standing current and future sampling gaps across the Americas
Source: Mem Inst Oswaldo Cruz. 2024 Sep 23;119:e230100. doi: 10.1590/0074-02760230100 (PMC11421423; doi:10.1590/0074-02760230100)
Supplement: Supplementary file 1 [file 1678-8060-mioc-119-e230100-s.pdf]

Citations for collections that we obtained occurrence data from at iDigBio: http://www.idigbio.org/portal (2022), 56 records, accessed on 2022-05-19T10:38:04.447177, contributed by 1 Recordsets, Recordset identifiers: http://www.idigbio.org/portal/recordsets/e4b33221-1e2c-405c-ac02-a39d93f9a69b (56 records).

TABLE I

A full synopsis of the pre-1999 model average area under the receiver-operator curve (AUC) and true skill statistic (TSS) scores

Species (# of occurrences)	Average AUC	Average TSS
Eratyrus cuspidatus (90)	0.97	0.88
Panstrongylus geniculatus (575)	0.90	0.67
Panstrongylus guentheri (74)	0.93	0.72
Panstrongylus lutzi (437)	0.92	0.72
Panstrongylus megistus (1,387)	0.94	0.77
Panstrongylus rufotuberculatus (136)	0.96	0.81
Paratriatoma hirsuta (111)	0.97	0.86
Rhodnius pallescens (86)	0.96	0.82
Rhodnius pictipes (51)	0.95	0.85
Rhodnius prolixus (748)	0.97	0.85
Triatoma brasiliensis	Not included	Not included
Triatoma delpontei (34)	0.96	0.82
Triatoma dimidiata (370)	0.98	0.85
Triatoma dispar (150)	0.98	0.97
Triatoma garciabesi (65)	0.95	0.87
Triatoma gerstaeckeri (249)	0.97	0.80
Triatoma guasayana (90)	0.95	0.86
Triatoma indictiva (50)	0.92	0.80
Triatoma infestans (455)	0.97	0.69
Triatoma lecticularia (35)	0.97	0.91
Triatoma melanocephala (82)	0.95	0.80
Triatoma patagonica (41)	0.95	0.75
Triatoma platensis (93)	0.91	0.80
Triatoma protracta (888)	0.91	0.66
Triatoma pseudomaculata (389)	0.95	0.72
Triatoma recurva (39)	0.95	0.82
Triatoma rubida (413)	0.96	0.79
Triatoma rubrofasciata (28)	0.93	1
Triatoma rubrovaria (70)	0.92	0.83
Triatoma sanguisuga (309)	0.97	0.70
Triatoma sordida (292)	0.99	0.69
Panstrongylus tibiamaculatus (30)	0.99	0.91
Triatoma vitticeps (86)	0.99	0.93

TABLE II

A full synopsis of the all-occurrence average area under the receiver-operator curve (AUC) and true skill statistic (TSS) scores

Species (# of occurrences)	Average AUC	Average TSS
Eratyrus cuspidatus (115)	0.93	0.765
Panstrongylus geniculatus(768)	0.84	0.537
Panstrongylus guentheri (99)	0.92	0.717
Panstrongylus lutzi (625)	0.93	0.727
Panstrongylus megistus (2,650)	0.94	0.777
Panstrongylus rufotuberculatus (192)	0.94	0.75
Paratriatoma hirsuta (119)	0.97	0.89
Rhodnius pallescens (111)	0.94	0.735
Rhodnius pictipes (83)	0.93	0.737
Rhodnius prolixus (958)	0.97	0.843
Triatoma brasiliensis (4,570)	0.94	0.762
Triatoma delpontei (58)	0.92	0.766
Triatoma dimidiata (1,564)	0.98	0.888
Triatoma dispar (257)	0.98	0.884
Triatoma garciabesi (238)	0.96	0.828
Triatoma gerstaeckeri (1,553)	0.96	0.825
Triatoma guasayana (340)	0.98	0.877
Triatoma indictiva (163)	0.97	0.811
Triatoma infestans (4,297)	0.96	0.805
Triatoma lecticularia (50)	0.98	0.894
Triatoma melanocephala (92)	0.96	0.814
Triatoma patagonica (132)	0.94	0.731
Triatoma platensis (175)	0.91	0.705
Triatoma protracta (1,192)	0.92	0.682
Triatoma pseudomaculata (1,370)	0.92	0.730
Triatoma recurva (79)	0.95	0.807
Triatoma rubida (542)	0.96	0.813
Triatoma rubrofasciata (30)	0.97	0.933
Triatoma rubrovaria (164)	0.93	0.850
Triatoma sanguisuga (1,421)	0.91	0.659
Triatoma sordida (1,410)	0.96	0.692
Panstrongylus tibiamaculatus (63)	0.99	0.936
Triatoma vitticeps (122)	0.99	0.909

TABLE III

A full synopsis of the pre-1999 model vs. range-map scores for each species across all time periods of inference. Values may range between 0 to 1 (with 1 indicating perfect agreement between expert range map and distribution model)

Species (# of occurrences)	Contemp.	2041-2060	2061-2080	2081-2100
Eratyrus cuspidatus (90)	0.38	0.42	0.46	0.47
Panstrongylus geniculatus (575)	0.24	0.27	0.28	0.29
Panstrongylus guentheri (74)	0.78	0.74	0.71	0.65
Panstrongylus lutzi (437)	0.82	0.77	0.71	0.64
Panstrongylus megistus (1,387)	0.91	0.87	0.84	0.82
Panstrongylus rufotuberculatus (136)	0.63	0.72	0.74	0.74
Paratriatoma hirsuta (111)	0.72	0.72	0.71	0.69
Rhodnius pallescens (86)	0.54	0.67	0.75	0.80
Rhodnius pictipes (51)	0.43	0.37	0.36	0.35
Rhodnius prolixus (748)	0.51	0.51	0.51	0.52
Triatoma brasiliensis	Not included	Not included	Not included	Not included
Triatoma delpontei (34)	0.53	0.53	0.53	0.49
Triatoma dimidiata (370)	0.83	0.77	0.76	0.73
Triatoma dispar (150)	0.67	0.75	0.78	0.77
Triatoma garciabesi (65)	0.78	0.77	0.75	0.73
Triatoma gerstaeckeri (249)	0.79	0.76	0.75	0.74
Triatoma guasayana (90)	0.88	0.83	0.80	0.75
Triatoma indictiva (50)	0.42	0.42	0.41	0.40
Triatoma infestans (455)	0.82	0.82	0.79	0.77
Triatoma lecticularia (35)	0.40	0.22	0.14	0.06
Triatoma melanocephala (82)	0.87	0.84	0.82	0.80
Triatoma patagonica (41)	0.69	0.66	0.64	0.58
Triatoma platensis (93)	0.75	0.73	0.70	0.66
Triatoma protracta (888)	0.68	0.67	0.67	0.67
Triatoma pseudomaculata (389)	0.68	0.76	0.75	0.76
Triatoma recurva (39)	0.46	0.43	0.42	0.41
Triatoma rubida (413)	0.88	0.80	0.75	0.67
Triatoma rubrofasciata (28)	0.65	0.73	0.75	0.77
Triatoma rubrovaria (70)	0.86	0.85	0.83	0.81
Triatoma sanguisuga (309)	0.64	0.57	0.55	0.54
Triatoma sordida (292)	0.76	0.66	0.62	0.59
Panstrongylus tibiamaculatus (30)	0.71	0.79	0.79	0.80
Triatoma vitticeps (86)	0.89	0.90	0.89	0.87

TABLE IV

A full synopsis of the all-occurrence model vs. range-map scores for each species across all time periods of inference. Values may range between 0 to 1 (with 1 indicating perfect agreement between expert range map and distribution model)

Species (# of occurrences)	Contemp.	2041-2060	2061-2080	2081-2100
Eratyrus cuspidatus (115)	0.46	0.57	0.60	0.62
Panstrongylus geniculatus(768)	0.29	0.31	0.31	0.32
Panstrongylus guentheri (99)	0.78	0.75	0.73	0.67
Panstrongylus lutzi (625)	0.82	0.78	0.23	0.68
Panstrongylus megistus (2,650)	0.92	0.88	0.86	0.84
Panstrongylus rufotuberculatus (192)	0.62	0.74	0.75	0.77
Paratriatoma hirsuta (119)	0.75	0.73	0.72	0.70
Rhodnius pallescens (111)	0.58	0.70	0.78	0.83
Rhodnius pictipes (83)	0.45	0.38	0.38	0.37
Rhodnius prolixus (958)	0.52	0.52	0.53	0.54
Triatoma brasiliensis (4,570)	0.82	0.69	0.58	0.47
Triatoma delpontei (58)	0.59	0.57	0.55	0.52
Triatoma dimidiata (1,564)	0.87	0.82	0.80	0.77
Triatoma dispar (257)	0.81	0.88	0.90	0.90
Triatoma garciabesi (238)	0.87	0.85	0.84	0.80
Triatoma gerstaeckeri (1,553)	0.89	0.87	0.87	0.87
Triatoma guasayana (340)	0.95	0.92	0.90	0.88
Triatoma indictiva (163)	0.58	0.56	0.55	0.56
Triatoma infestans (4,297)	0.89	0.88	0.86	0.84
Triatoma lecticularia (50)	0.46	0.29	0.20	0.10
Triatoma melanocephala (92)	0.88	0.85	0.85	0.83
Triatoma patagonica (132)	0.82	0.78	0.76	0.72
Triatoma platensis (175)	0.82	0.79	0.76	0.70
Triatoma protracta (1,192)	0.72	0.70	0.70	0.70
Triatoma pseudomaculata (1,370)	0.69	0.76	0.77	0.78
Triatoma recurva (79)	0.60	0.50	0.48	0.48
Triatoma rubida (542)	0.90	0.83	0.79	0.73
Triatoma rubrofasciata (30)	0.57	0.64	0.67	0.69
Triatoma rubrovaria (164)	0.90	0.89	0.88	0.86
Triatoma sanguisuga (1,421)	0.76	0.68	0.66	0.64
Triatoma sordida (1,410)	0.85	0.77	0.73	0.68
Panstrongylus tibiamaculatus (63)	0.78	0.82	0.82	0.83
Triatoma vitticeps (122)	0.90	0.88	0.85	0.82

TABLE V

Phylogenetic signal metrics for the pre-1999 and all occurrence analysis of expert range map scores across all time periods as well as the difference in scores from now into 2100. Asterisks indicate significance at $p < 0.05$

Metric	Bloomberg's K	Bloomberg's K-star	Pagels lambda
PRE-1999			
Contemporary score	0.259	0.306	0.306
2041 - 2060 score	0.239	0.289	0.198
2061 - 2080 score	0.236	0.292	0.163
2081 - 2100 score	0.213	0.285	0.068
Shift in score	0.399	0.582*	6.19×10^{-5}
All occurrence			
Contemporary score	0.312	0.328	0.349*
2041 - 2060 score	0.270	0.282	0.274
2061 - 2080 score	0.257	0.275	0.239
2081 - 2100 score	0.231	0.266	0.178
Shift in score	0.355	0.537	4.52×10^{-5}
